# Indication to Open Anatrophic Nephrolithotomy in the Twenty-First Century: A Case Report

**DOI:** 10.1155/2012/851020

**Published:** 2012-11-22

**Authors:** Alfredo Maria Bove, Emanuela Altobelli, Maurizio Buscarini

**Affiliations:** Department of Urology, Campus Bio-Medico University, Alvaro Del Portillo Street, 00128 Rome, Italy

## Abstract

*Introduction*. Advances in endourology have greatly reduced indications to open surgery in the treatment of staghorn kidney stones. Nevertheless in our experience, open surgery still represents the treatment of choice in rare cases. *Case Report*. A 71-year-old morbidly obese female patient complaining about occasional left flank pain, and recurrent cystitis for many years, presented bilateral staghorn kidney stones. Comorbidities were obesity (BMI 36.2), hypertension, type II diabetes, and chronic obstructive pulmunary disease (COPD) hyperlipidemia. Due to these comorbidities, endoscopic and laparoscopic approaches were not indicated. We offered the patient staged open anatrophic nephrolithotomy. *Results*. Operative time was 180 minutes. Blood loss was 500 cc. requiring one unit of packed red blood cells. Hospital stay was 7 days. The renal function was unaffected based on preoperative and postoperative serum creatinine levels. Stone-free status of the left kidney was confirmed after surgery with CT scan. *Conclusions*. Open surgery can represent a valid alterative in the treatment of staghorn kidney stones of very selected cases. A discussion of the current indications in the twenty-first century is presented.

## 1. Introduction

Surgical management of nephrolithiasis has changed dramatically in the last few decades. While previously, the majority of patients required an open surgical approach, today less invasive procedures, such as extracorporeal shock waves lithotripsy (ESWL), ureterorenoscopy (URS), and percutaneous nephrolithotripsy (PNL), have promoted a rapid decrease of the use of open surgery for both ureteral and renal stones [1, 2]. The subsequent introduction of laparoscopic approach has almost eliminated the need for open operations in the treatment of renal and ureteral stones. Laparoscopy is needed in complex staghorn stones that would necessitate multiple, simultaneous or subsequent, percutaneous renal accesses [3–10]. Even if anatrophic nephrolithotomy is currently performed laparoscopically, in patients affected by severe cardiac or pulmonary diseases or with a previous laparotomy, laparoscopic approach may not be indicated. In the era of mininvasive treatments, laparotomy is rarely required, but it is important to recognize patients in whom open anatrophic nephrolithotomy could represent a valid choice of treatment [11]. This paper presents one of such patients as well as a discussion of the modern indications for this technique.

## 2. Case Report

**Figure 1:**
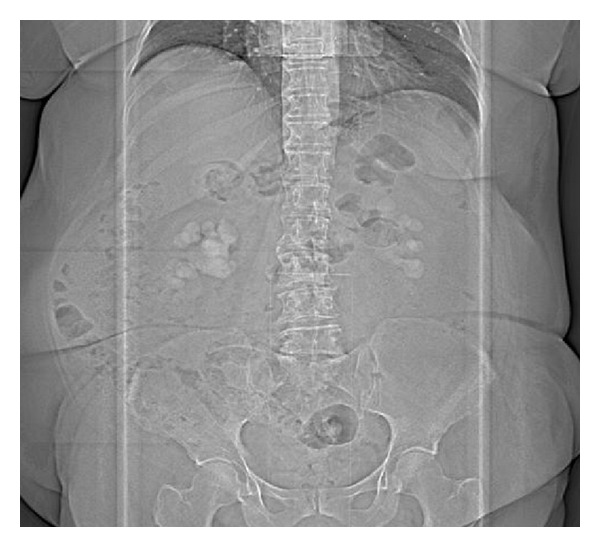
Abdominal X-ray.

A 71-year-old female patient with a BMI of 36,2 was referred from General Medicine Department with a diagnosis of bilateral staghorn kidney stone, documented by an abdominal X-ray (Figure 1). She complained about occasional left flank pain and recurrent cystitis from many years.

The patient was also affected by hypertension, diabetes type II, chronic obstructive pulmonary disease (COPD), and IIb hyperlipidemia. Renal function was preserved with a serum creatinine of 1.06 mg/dL. She had undergone laparotomic cholecystectomy and appendectomy 25 years previously and reported a previous left ESWL 8 years earlier. 

**Figure 2:**
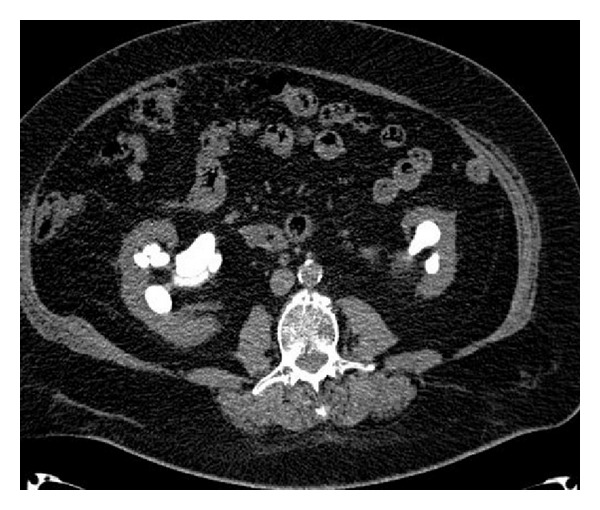
Preoperative CT scan.

**Figure 3:**
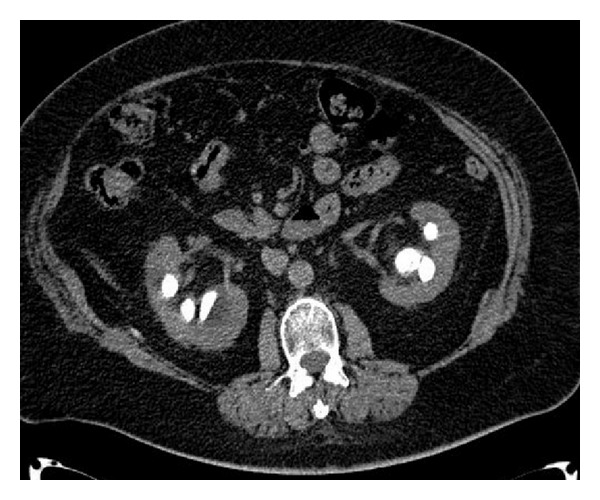
Preoperative CT scan.

During the hospitalization, an abdominal CT scan was performed and confirmed the presence of bilateral sthagorn stones (Figures 2 and 3). Urine culture was positive for E. coli growth and a specific antibiotic therapy with intravenous Cefazolin infusion was prescribed for 7 days preoperatively.

A percutaneous treatment was impractical due to stones volume and complexity, obesity, and low compliance of patient for possible need of repeat treatments. A laparoscopic approach was not indicated because of the severe long standing COPD and previous open abdominal operations.

For these reasons the patient was scheduled for staged open bilateral anatrophic nephrolithotomy, and we elected to treat the side with the lower stone burden first.

## 3. Surgical Technique

The kidney was exposed through a flank incision on the 11th intercostal space. When access was gained into the retroperitoneal space, Gerota's fascia was longitudinally incised and perinephric fat carefully dissected off the entire renal capsule. The renal artery and vein were identified and the posterior segmental artery was isolated and temporarily clamped during the intravenous injection of 20 mL of methylene blue to identify Brodel's line. The main renal artery and vein were then occluded with bulldog clamps. and cold ischemia was performed surrounding the kidney with ice slush. Dry laparotomy sponges were used to pack away the peritoneal contents.

**Figure 4:**
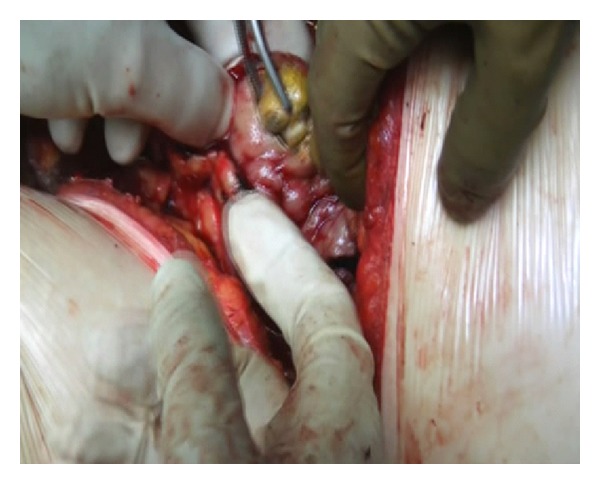
Calculi extraction.

**Figure 5:**
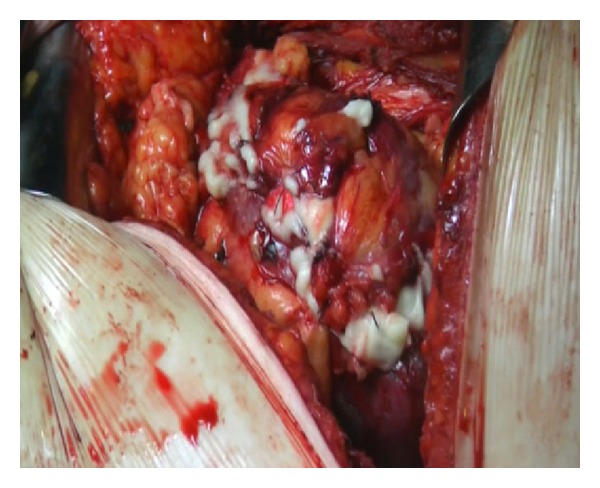
Floseal application.

**Figure 6:**
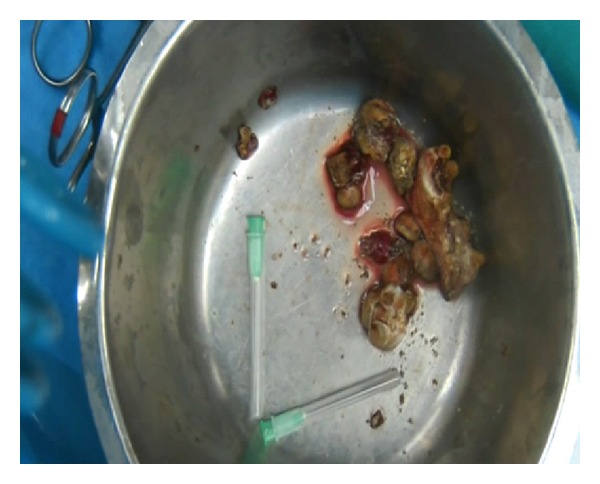
Stones removed.

Slush was applied as needed throughout the case to maintain adequate regional hypothermia, while the renal vessels were occluded. A nephrotomy was made along the previously defined anatrophic plane. The collecting system was opened and the stones were exposed. Staghorn calculi were completely removed (Figure 4) and the absence of stones was evidenced with intraoperative fluoroscopy. A doble J 6 Fr. Standard Multilenght stent was placed in the ureter, and a Malecot tube was left in pelvis. A calicoplasty was performed with a 6–0 Vicryl suture and parenchymal suture was completed with a 3–0 Vicryl suture. Floseal was applied on the suture line (Figure 5). A 24ch drainage was left in the perirenal space. Stone weight was 150 grams (Figure 6).

During the procedure, blood loss was 500 mL, and cold ischaemia time was 30 minutes. Total operative time was 180 minutes. An intraoperative blood transfusion was required.

## 4. Results

**Figure 7:**
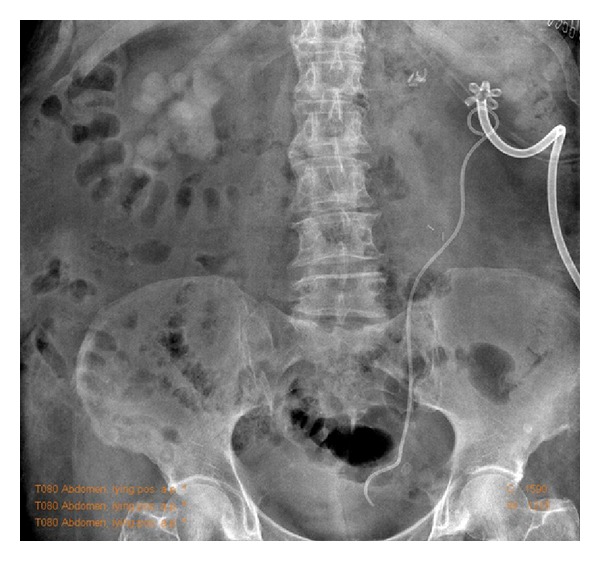
Postoperative abdominal X-ray.

**Figure 8:**
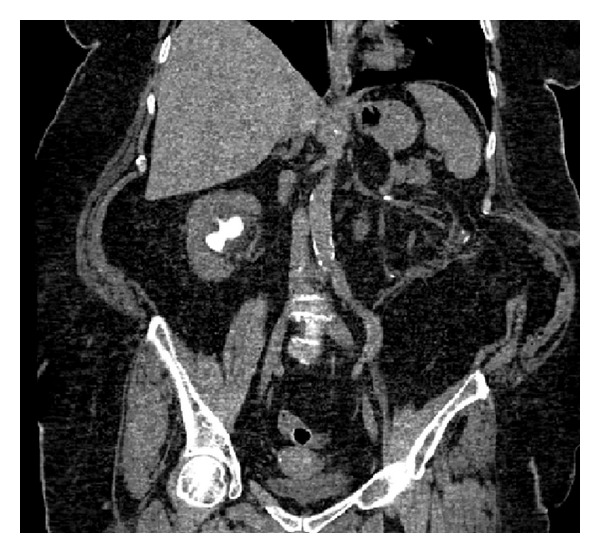
Postoperative CT scan.

**Figure 9:**
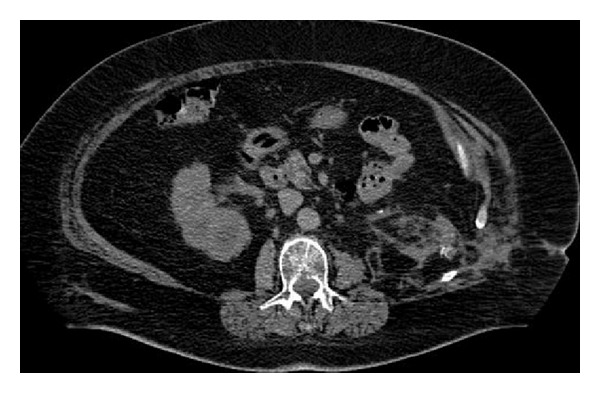
Postoperative CT scan.

The abdominal X-ray performed 5 days after surgery confirmed the left kidney to be stone-free. The drain was removed on POD 5. Ureteral stent was removed after 3 weeks. After 3 months, a renal ultrasound showed no hydronephrosis, no left kidney calculi, and the persistence of right kidney staghorn calculi with a sufficiently represented parenchyma. The CT scan performed 9 months after surgery confirmed a stone-free left kidney (Figures 7, 8, and 9). Normal renal function was assessed. Urine culture at one month was positive for Klebsiella Pneumoniae and antibiotic therapy with Ceftriaxone was prescribed until complete remission, at 6 month and at one year after surgery were negative. Patient sank into a deep depression after her husband's death, and for this reason she refused second stage procedure on right kidney.

## 5. Discussion

In the last few decades, surgical approach to stone treatment has changed dramatically. ESWL and Endoscopic treatment have virtually eliminated the need for open surgery in kidney and ureteral stones [1, 2]. The advent of laparoscopic stone removing procedures has further reduced the need to perform open surgery, even anatrophic nephrolithotomy [3–10]. The great limitation of laparoscopic surgery resides in patients' comorbid conditions such as: severe heart failure, severe pulmonary disease like COPD, previous laparotomy. According to European association of urology guidelines (EAU 2012 guidelines), the most common indications for open surgery are failure of ESWL and/or PNL, or URS; intrarenal anatomical abnormalities such as infundibular stenosis, stone in the calyceal diverticulum (particularly in an anterior calyx), obstruction of the ureteropelvic junction, stricture if endourologic procedures have failed or are not promising; obesity; skeletal deformity such as contractures and fixed deformities of hips and legs; concomitant open surgery; nonfunctioning lower pole when partial nephrectomy is indicated or nonfunctioning kidney where nephrectomy is required [11–15]. According to these indications, our rate of open procedures was 1 on 500 cases of stones treatment, almost 1 per year, for a total of 6 cases in the last 5 years.

It is essential to carefully select the patient for this treatment, frequently they may prefer a single procedure avoiding the risk of a repeated PNL [16]. 

For paediatric population, in contrast with adults, nephrolithotomy is still considered a treatment of choice because it allows, through a small access, the best chance of stone-free rate for staghorn and complex kidney stones, with a safe reconstruction of the renal collecting system [17–20].

Morbidly obese patients may require this approach as their body habitus precludes fluoroscopic imaging and endoscopic manoeuvring required for PNL.

Patients with staghorn calculi in a nonfunctioning kidney are candidates for nephrectomy, and the procedure also may be considered if the stone-laden kidney has irrevocably poor function providing the contralateral renal unit has satisfactory function. Laparoscopic nephrectomy is an option, but open surgical nephrectomy may be a safer approach if there is intense perirenal inflammation, such as that which occurs with chronic xanthogranulomatous pyelonephritis [21–24]. For these reasons open anatrophic nephrolithotomy represented the treatment of choice for our morbidly obese patient affected by COPD. The aim of the procedure was to remove all calculi and fragments, improving urinary drainage, eradicating infections, and preserving renal function. With this approach, we obtained a stone-free left kidney without significant blood loss and with few days of hospitalisation. A retroperitoneal approach, packing away the peritoneal contents with dry laparotomy sponges allows to perform a safe cold ischemia, from 5° to 20°C range, surrounding the kidney with ice slush, and preventing frost ischemic bowel damage. This represents the major advantage of retroperitoneal approach compared to transperitoneal approach. In expert hands, anatrophic nephrolithotomy is an effective procedure, which spares renal function. Our rate of open stone procedures is obviously lower than our endoscopic one, but likely higher than reported stones patients population, mostly attributable to the significant number of complex stone cases that we see in our centre, including many cases specifically referred to our institution for possible open surgery. Because of this probable bias, our observed rate of open surgery should not be interpreted as indicative of the general stone patient population, although it does allow for the examination of common indications for open surgery [11].

## 6. Conclusions

Open stone surgery continues to represent a reasonable alternative for a small segment of the urinary stone population. In our experience, open surgery still plays a role in the treatment of staghorn stone disease, even if rarely required. Open surgical approach appears necessary in minimally invasive treatment failures. In our experience the impossibility to perform a laparoscopic approach represents the most common indication for open surgery. In particular older patients or affected by many comorbidities. In these selected cases open surgery may be performed with high stone-free rate and very low morbidity.
